# Antihypertensive Peptides and Hydrolysates Derived from Plant Proteins and Their Bioavailability

**DOI:** 10.3390/foods15050900

**Published:** 2026-03-05

**Authors:** Seyi David Adebayo, Sukanya Poddar, Jianmei Yu

**Affiliations:** Department of Family and Consumer Sciences, North Carolina Agricultural and Technical State University, Greensboro, NC 27411, USA

**Keywords:** antihypertensive, plant protein-derived peptides/hydrolysates, ACE inhibition, renin inhibition, bioavailability

## Abstract

Hypertension is a major controllable risk factor associated with cardiovascular disease, myocardial infarction, stroke, heart failure, and end-stage diabetes. While commercial antihypertensive drugs are effective in managing high blood pressure, they often come with a range of side effects. Additionally, individuals who begin anti-hypertensive treatment may need to continue these medications throughout their lifetime. In response to these challenges, recent studies have focused on the potential of antihypertensive peptides and hydrolysates derived from food proteins. Food protein-derived peptides and hydrolysates help lower blood pressure (hypertension) primarily by inhibiting the renin–angiotensin system (RAS). Some peptides or protein hydrolysates derived from milk and fish have been proven to be safe and effective anti-hypertensive products, and they are currently on the market. The bioactive peptides and hydrolysates derived from plant proteins with a long history of safe consumption are generally considered safe and have shown some advantages over animal protein-derived peptides. This review provides an up-to-date overview of plant protein-derived antihypertensive peptides and hydrolysates, covering their ACE- and renin-inhibiting activities and mechanisms, in vivo and clinical evidence, bioavailability, production and commercialization challenges, and perspectives for future research.

## 1. Introduction

Hypertension is a risk factor for both cardiovascular disease (CVD) and overall mortality. According to the World Health Organization (WHO), hypertension affects 1 in 3 adults worldwide and is the leading cause of CVD, including stroke and heart attack, kidney damage, and many other health problems, and is a leading cause of premature death [1]. In the United States, the prevalence of adult hypertension reached 47.7% during the period of August 2021 to August 2023 [2]. In 2024, the Centers for Disease Control and Prevention (CDC) reported that heart disease was the top cause of death in the U.S., responsible for around 919,032 deaths, equivalent to one in three deaths [3]. The economic burden is equally staggering: annual healthcare costs of cardiovascular risk factors are projected to triple between 2020 and 2050, rising from $400 billion to $1.34 trillion. Healthcare costs for cardiovascular conditions specifically are projected to nearly quadruple from $393 billion to $1.49 trillion, while productivity losses are expected to increase by 54%, from $234 billion to $361 billion [4].

The renin–angiotensin–aldosterone system (RAAS) is a primary pathway in the development of hypertension, and renin and angiotensin-converting enzyme (ACE) are pivotal enzymes in this system. When renal perfusion pressure drops, juxtaglomerular cells release renin. This enzyme cleaves angiotensinogen to produce angiotensin I. ACE then converts angiotensin I to angiotensin II, a potent vasoconstrictor that increases blood pressure by inducing arteriolar constriction, promoting sodium reabsorption, and stimulating aldosterone secretion [5].

Current synthetic antihypertensive drugs, including ACE inhibitors (captopril, lisinopril, enalapril) and the only FDA-approved renin inhibitor, Aliskiren, effectively manage hypertension but cause undesirable side effects, including hypotension, cough, hyperkalemia, and renal impairment [6]. These limitations have driven the exploration of safe, cost-effective antihypertensive peptides from food sources [7].

Various ACE- and renin-inhibitory peptides have been identified in food proteins and protein hydrolysates from milk, fish, meat, legumes, and oilseeds, including flaxseed and hemp [8,9]. ACE-inhibitory peptides are already commercially available in various functional foods and fermented products, and clinical trials have demonstrated their blood pressure-lowering efficacy [10]. While regulatory pathways for specific health claims continue to evolve, the existing Generally Recognized as Safe (GRAS) status of bioactive peptides provides a clear pathway for market entry and product development of plant-derived antihypertensive peptides. The peptides and hydrolysates are considered GRAS only if the protein raw material, proteolytic enzymes, microorganisms, and all materials used in the production of the peptides are food-grade materials under U.S. federal regulations [11].

The aim of this comprehensive review is to provide up-to-date information on plant protein-derived antihypertensive peptides and hydrolysates, including their in vitro ACE- and renin-inhibitory activities, in vivo and clinical evidence, bioavailability, production and commercialization challenges, and directions for future research. An extensive literature search was conducted across ScienceDirect, Google Scholar, and PubMed using the Boolean search strategy: (“plant protein hydrolysates” OR “plant-derived peptides” OR “plant protein-derived peptides”) AND (“ACE inhibition” OR “angiotensin converting enzyme inhibition” OR “renin inhibition”) AND (“hypertension” OR “antihypertensive”). The search was limited to peer-reviewed articles published in English between 2022 and 2026. Although the search was primarily limited to this period, seminal and foundational studies on renin-inhibitory peptides and in vivo antihypertensive activity published as far back as 2013 were included where relevant, as the literature in these areas remains limited and earlier studies provide important foundational context.

## 2. Hypertension: Classification, Prevalence, and Management

### 2.1. Classification

Hypertension is diagnosed when systolic blood pressure (SBP) ≥140 mmHg and/or diastolic blood pressure (DBP) ≥90 mmHg after repeated assessments [12,13]. Hypertension is classified into stages based on average blood pressure levels as shown in Table 1.

### 2.2. Global Prevalence and Disease Burden

An estimated 1.28 billion adults aged 30–79 worldwide have hypertension, with two-thirds living in low- and middle-income countries. Alarmingly, 46% are unaware of their condition, and fewer than half are diagnosed or treated [1]. Upper middle-income countries carry the largest burden at 506 million (44.2%), followed by lower middle-income countries at 443.8 million (38.7%), high-income countries at 134.1 million (11.7%), and low-income countries at 61.5 million (5.4%) [15]. Elevated blood pressure significantly increases the risk of cardiovascular mortality. In 2015, 10.7 million deaths were linked to elevated SBP, driven largely by ischemic heart disease and stroke, alongside a twofold rise in fatalities across East, South, and Southeast Asia and sub-Saharan Africa [16]. Hypertension is also a strong independent risk factor for chronic kidney disease and end-stage renal disease (ESRD). Stage 3 hypertensive patients show a 22-fold higher ESRD risk [17].

### 2.3. Causes of Hypertension

Blood pressure is influenced by non-modifiable factors such as age, sex, and genetics as well as modifiable lifestyle-related risk factors [18]. BP rises with age in both sexes, with men having higher levels at younger ages and women showing steeper increases per decade [19]. Disparities in hypertension prevalence across racial and ethnic groups have been documented globally and in the US, and are attributed to the interplay of genetic, social, cultural, and environmental factors [2,20,21]. Key modifiable risk factors include physical inactivity, smoking, excessive alcohol consumption, and dietary imbalances [22]. High sodium intake (>5 g/day) elevates BP through changes in vascular resistance and microvascular inflammation [23], while adequate potassium intake supports blood pressure regulation by promoting peripheral vasodilation and sodium excretion [24]. Physical inactivity increases hypertension risk by 30–50%, and a meta-analysis of 54 trials showed that aerobic exercise reduces SBP by 3.84 mmHg and DBP by 2.58 mmHg [25]. Obesity independently disrupts renal natriuresis by increasing sodium reabsorption and activating the renin–angiotensin and sympathetic nervous systems [26].

### 2.4. Management of Hypertension

Lifestyle modification is the first-line approach for blood pressure management. This approach includes weight reduction [27], sodium restriction [28], increased potassium intake [29], regular physical activity [25], reduced alcohol consumption [30], and the DASH diet. The DASH diet is most effective when combined with sodium restriction [27,28]. When lifestyle changes are insufficient at stage 1 or 2, pharmacological intervention is indicated. The American College of Cardiology/American Heart Association (ACC/AHA) guidelines recommend treatment to a blood pressure of 130/80 mmHg or higher in high-risk patients and 140/90 mmHg or higher in all patients [14]. First-line therapies include ACE inhibitors, ARBs, calcium channel blockers, and diuretics [6], with beta-blockers used as adjunct therapy in heart failure or post-myocardial infarction [31]. Aliskiren, a renin inhibitor, lowers BP and suppresses plasma renin activity when combined with thiazide diuretics, ACE inhibitors, or ARBs, though it is contraindicated with ACE inhibitors or ARBs in patients with diabetes [32,33]. While commercial antihypertensive medications are effective in managing high blood pressure, they often come with a range of side effects, such as dizziness, fatigue, and headaches that can impact patients’ compliance and quality of life. Additionally, individuals who begin antihypertensive treatment may need to continue these medications throughout their lifetime. The mechanisms of action and side effects of these drugs are detailed in Table 2.

In response to these challenges, studies have focused on the potential of antihypertensive peptides derived from food sources in past two to three decades. These natural peptides offer a promising alternative with potentially fewer or no side effects. This paves the way for innovative and more sustainable approaches to hypertension management. Based on European guidelines for hypertension management, the use of nutraceuticals may be a suitable option for patients with slightly elevated blood pressure, and can be used in combination with antihypertensive drugs to treat moderate hypertension [36].

## 3. Antihypertensive Bioactive Peptides and Hydrolysates

Renin and ACE are key enzymes of the renin–angiotensin–aldosterone system (RAAS), which plays a critical role in the development of hypertension. Renin, an enzyme from the kidneys, initiates the process by converting angiotensinogen to angiotensin I. ACE then converts angiotensin I to angiotensin II, a hormone that constricts blood vessels and increases blood pressure [5,37]. Therefore, in most studies of food protein-derived antihypertensive peptides and hydrolysates, ACE-inhibitory and renin-inhibitory activities have been used as indicators of antihypertensive potential.

In recent years, studies have shown that certain peptides found in food proteins have antihypertensive effects both in vitro and in vivo [38]. The first ACE inhibitory peptide was discovered in the venom of the Jararaca snake [39]. Since then, antihypertensive peptides have been isolated from various sources, including animals, plants, and microbes [40]. Antihypertensive peptides are specific peptides that, when released from their parent proteins, have the capacity to regulate blood pressure in the body by modulating the renin-angiotensin system [41]. A single, purified peptide might have a more potent or longer-lasting effect than a protein hydrolysate because it has a more defined structure that can be tailored to a specific mechanism, such as ACE inhibition and/or renin inhibition. Conversely, hydrolysates are unpurified mixtures of peptides produced by enzymatic hydrolysis of parent proteins and can offer broader or complementary benefits, although interactions and overall efficacy can be less predictable [42,43]. These peptides and hydrolysates have the potential to reduce the costs of medication needed to treat hypertension, a chronic ailment. Additionally, they do not cause the side effects of synthetic hypertension drugs [7] and can be economically produced from protein-rich by-products, reducing waste [44].

### 3.1. Production of Antihypertensive Peptides and Hydrolysates

The production of specific antihypertensive peptides requires steps like protein extraction, enzymatic or chemical hydrolysis, or fermentation to break down proteins into smaller peptides, fractionation by ultrafiltration, and purification by different preparative chromatography techniques (such as size exclusion chromatography, immobilized metal-affinity chromatography, ion exchange chromatography, and/or reversed-phase HPLC [45,46]. However, across these processes, a recurring limitation is that conventional peptide production techniques struggle to balance high specificity and yield with industrial scalability and cost-effectiveness.

#### 3.1.1. Protein Source

The production of bioactive peptides has traditionally relied on animal proteins, such as milk, meat, eggs, fish, and gelatin [47]. However, increasing environmental concerns and sustainability considerations have driven a shift toward plant sources. This transition also reflects the nutritional versatility of plants, which provide beneficial bioactive compounds including antioxidants, vitamins, and fiber. Plant proteins are cost-effective, readily available, nontoxic, and multifunctional. Plant-derived bioactive peptides are suitable for diverse consumer populations regardless of dietary restrictions [48]. Plant bioactive peptides can be obtained from a variety of sources, such as legumes, cereals, nuts, oilseed meals from soybean, peanuts, hemp seed, sesame seed, chia seed, pumpkin seeds, sunflower seeds, fruits, and vegetables [48,49,50,51]. The use of plant by-products can also be maximized. For example, El-Adawy & Taha showed that pumpkin oil extraction residues contain up to 70% protein [52]. Similarly, Yu et al. (2007) reported that defatted peanut residue could attain a protein content exceeding 50% [53].

#### 3.1.2. Protein Extraction

The conventional methods, such as alkaline extraction and isoelectric precipitation, are widely used but often compromise protein functionality, yield inadequately, and generate substantial waste [53,54]. Emerging approaches, including ultrasound-assisted extraction, pulsed electric fields, and subcritical water extraction, can enhance extraction efficiency and reduce environmental impact; however, their industrial adoption remains limited by high capital costs and process scalability constraints [55].

#### 3.1.3. Protein Hydrolysis

The production of antihypertensive peptides and hydrolysates from whole proteins leverages proteolytic enzymes’ inherent ability to cleave proteins. The most common enzymes used are digestive enzymes (such as pepsin, trypsin, and chymotrypsin) [56], microbial enzymes (such as Alcalase, Neutrase, and Flavourzyme from bacteria and fungi) [57,58], and plant-derived enzymes (such as papain, bromelain, and ficin) [59]. Fermentation is another method in which microorganisms, such as lactic acid bacteria (*Lactobacillus helveticus*, *Lactobacillus plantarum*), yeasts (*Saccharomyces cerevisiae*), or fungi (*Aspergillus oryzae*), produce proteolytic enzymes as they multiply [60]. This approach is cost-effective because it does not require purified enzymes, and it has the added benefit of generating both bioactive peptides and probiotic microorganisms. Common fermented foods like yogurt, kefir, cheese, miso, and tempeh contain naturally occurring antihypertensive peptides. Compared to enzymatic hydrolysis, fermentation may be less reproducible and requires more frequent monitoring. However, both methods are bioprocesses susceptible to variations in factors such as pH, temperature, and reaction time, which can affect the final product profile [60]. Chemical hydrolysis (acid or alkali) is rarely used due to poor specificity, harsh conditions, and potential degradation of nutritional quality [61].

Alcalase is perhaps the most widely used enzyme for antihypertensive peptide production, owing to its broad cleavage specificity and capacity to achieve high degrees of hydrolysis in relatively short timeframes, generating small peptides that are more readily absorbed and have been associated with greater ACE inhibitory potency [57,62]. This is illustrated by some studies on plant proteins, where Alcalase consistently produced higher ACE inhibitory activity compared to other proteases such as Savinase, Protamex, Corolase 7089, papain and trypsin across hydrolysis times from 1 to 6 h [63,64]. However, in some cases, alternative enzymes or combinations outperform it. For example, Thermolysin hydrolyzed cherry stone protein yielded hydrolysates with higher antioxidant and antihypertensive capacities than Alcalase [65]. Enzyme selection is therefore largely dictated by the target substrate. The parent protein’s primary amino acid sequence and three-dimensional structure determine which bioactive peptides are encrypted within the protein and accessible to proteolytic cleavage [66]. Proteins with compact, highly cross-linked structures may require pretreatment or denaturation to enhance enzyme accessibility [67].

For a given protease and protein substrate, the antihypertensive potential of the hydrolysate and the yield of specific peptides vary with hydrolysis conditions, such as enzyme-to-substrate ratio, enzyme concentration, pH, temperature, and hydrolysis time. Although the optimal pH and temperature ranges of commercially available proteases are usually known (often provided by the manufacturers), fine-tuning is needed for specific substrates [68] because protease activity and stability are strongly influenced by pH and temperature. The optimal duration is consequently substrate- and enzyme-specific. The enzyme-to-substrate ratio and hydrolysis time at the optimal pH and temperature need to be optimized for the specific protease and substrate using degree of hydrolysis and ACE inhibitory activity as response indicators [69]. While increasing hydrolysis time initially promotes greater peptide bond cleavage and the release of bioactive peptides, prolonged proteolysis can degrade active sequences into smaller inactive fragments, causing ACE inhibitory activity to plateau or decline. In addition, the presence of inorganic salts, co-solvents, and other additives in the reaction medium can further affect both the hydrolysis process and the properties of the resulting peptides [70,71,72]. While certain additives, such as surfactants, are deliberately introduced to enhance enzyme-substrate interactions and improve peptide release [70], inorganic salts have been shown to interfere with peptide structure and complicate downstream bioactivity assessments and are therefore typically removed during preparation [72].

#### 3.1.4. Separation and Purification

Once hydrolysis is complete, fractionation and purification are required to isolate peptides with the highest antihypertensive potential. Ultrafiltration is used to obtain fractions containing peptides with higher antihypertensive potential [57], but membrane fouling and low selectivity lead to reduced flux and yield, necessitating frequent cleaning and regeneration [73]. To obtain a specific peptide, further separation by chromatographic methods followed by activity tests is required, but these are resource-intensive, time-consuming, and require large volumes of solvent and specialized equipment [74,75]. Yield losses at each purification step can also be substantial, further driving up costs [75]. In essence, the complexity of peptide purification results in high costs and low yields, making its use in nutraceuticals and functional foods challenging.

While specific peptides often exhibit higher ACE-inhibitory activity, protein hydrolysates may offer a more practical approach. Hydrolysates are relatively inexpensive to produce, often requiring only the incubation of food proteins with food-grade enzymes or microorganisms under optimized conditions, followed by separation of solid and liquid by filtration or centrifugation, and/or freeze-drying, with no further purification steps [76]. This reduces production costs and minimizes processing, aligning with consumer preferences for clean-label and minimally processed foods. While batch-to-batch variability can occur when hydrolysis conditions are not carefully controlled, standardized protocols and quality control measures enable reproducible hydrolysate production at a commercial scale. In addition, several studies have reported comparable or even superior ACE-inhibitory activity (lower IC_50_ values) in crude hydrolysates compared to their purified fractions [77,78,79]. This may be due to additive or complementary effects among peptides in the hydrolysate, in which multiple peptides contribute to antihypertensive potential through distinct mechanisms.

### 3.2. ACE-Inhibitory Activity of Plant Protein-Based Peptides and Hydrolysates

#### 3.2.1. Categories of ACE-Inhibitory Peptides

ACE inhibitory peptides, regardless of their source, can be categorized into three types based on their mechanism of action:

1. Inhibitor Type: These peptides maintain their inhibitory activity without structural modification after interaction with ACE. IY (IC_50_ = 2.1 μM) [80].
2. Prodrug Type: These peptides are initially inactive but become more potent after being cleaved by proteases in the serum, gastrointestinal tract, or ACE itself. For example, LKPNM (IC_50_ = 2.4 μM) is hydrolyzed by ACE to produce LKP (IC_50_ = 0.32 μM), which has 8 times higher ACE-inhibitory activity [81].
3. Substrate Type: These peptides act as ACE substrates and are cleaved into fragments with reduced or no inhibitory activity. FKGRYYP (IC_50_ = 0.55 μM) is hydrolyzed by ACE to FKG, RY, and YP (IC_50_ = 34 μM) [82].

Understanding these categories is important for predicting the stability and effectiveness of peptides both in vitro and in vivo. Table 3 presents studies of ACE-inhibiting peptides from plant sources since 2020. For a broader overview, readers are referred to excellent previous review articles [9,40,83,84,85]. Several peptides in Table 3 have high in vitro potency but have not been tested in vivo using animal or human models. Quinoa-derived SAPPP had an IC_50_ of 510 µg/mL [86] but lacks in vivo validation. Similarly, broccoli-derived KSVLLKF (IC50 = 0.129 μg/mL) [87] and camellia seed-derived VVVPQN (IC_50_ = 130 μg/mL) [88] exhibit strong in vitro activity without subsequent physiological testing.

#### 3.2.2. Molecular Mechanisms of ACE Inhibition of Plant-Derived ACE-Inhibitory Peptides

Among the various mechanisms by which food-derived peptides exert antihypertensive effects, ACE inhibition is the most studied. Bioactive peptides suppress ACE activity through either competitive or non-competitive binding [111]. For example, Shi et al. (2014) reported that peanut-derived peptides competitively bind to the active site of ACE [112]. Food-derived ACE-inhibitory peptides exert their effects through specific structural interactions with the enzyme. At the N-terminus, branched amino acid residues (N1, N2, N3) interact with ACE’s stabilizing residues and the Zn^2+^ cofactor, creating a hydrophobic shield that prevents water attack and restricts substrate access to the catalytic site [113]. Structure–activity relationships reveal that C-terminal regions are equally critical for ACE binding [114]. ACE shows a strong preference for peptides with hydrophobic amino acids (aromatic or branched side chains) and basic amino acids at the C-terminal [115,116]. Residues such as Tyr, Phe, Trp, Pro, His, and Arg provide particularly potent ACE inhibition, with stronger binding interactions at the active site explaining their enhanced effectiveness [117,118]. Additionally, ACE requires L-configuration at the third position from the C-terminus [119], and given that proline residues are far more likely to adopt the cis configuration than other amino acids, with trans being only approximately four times more dominant at proline bonds compared to 1000-fold for other peptide bonds [120], cis/trans isomerization at C-terminal Pro residues represents a particularly significant geometric determinant of ACE binding affinity, with the cis configuration demonstrated to be the more potent geometric form for ACE inhibition [121].

The antihypertensive effectiveness of these peptides depends on their chain length and amino acid composition, which is why fractionation techniques are used to separate hydrolysate peptides based on molecular weight, hydrophobicity, or net charge. Most identified ACE-inhibiting peptides are relatively small, consisting of 2–20 amino acid residues with low molecular weights [44]. Studies have consistently shown an inverse relationship between molecular weight and ACE-inhibitory activity. For example, Wu & Ding (2001) found that ACE inhibition in soy protein hydrolysate increased as peptide size decreased [113], while Ariza-Ortega et al. (2014) reported that larger peptides from *Phaseolus vulgaris* had reduced ACE-inhibition [122].

### 3.3. Plant-Derived Renin-Inhibitory Peptides

Renin represents another key target in the renin–angiotensin system for blood pressure regulation. Several studies have identified renin-inhibitory properties of plant protein hydrolysates from diverse sources, including peanuts [57], rapeseed [76], African yam bean seed [77], and hemp seed [79]. However, renin inhibition research remains limited compared to ACE inhibition studies, and available evidence indicates that peptides generally exhibit weaker renin-inhibitory activity than ACE-inhibitory activity [79,123]. This pattern suggests that most food protein hydrolysates target ACE more readily than renin in the cardiovascular regulatory pathway. In hypertensive states, increased vascular resistance and pressure stimulate the kallikrein–kinin system, leading to enhanced bradykinin release as a compensatory mechanism to promote vasodilation and reduce blood pressure [124]. However, ACE rapidly degrades bradykinin while also generating the vasoconstrictor angiotensin II, limiting bradykinin’s protective effect [124]. Since inhibiting renin alone does not prevent ACE from degrading bradykinin, combined inhibition of both enzymes is considered more effective for managing hypertension, as it reduces angiotensin II levels while preserving bradykinin’s vasodilatory action [125].

#### 3.3.1. Mechanisms of Renin Inhibition

Renin-inhibitory peptides exert their antihypertensive effects through multiple mechanisms. At the molecular level, peptides can directly inhibit renin through competitive, noncompetitive, or mixed-type inhibition. For example, peptides from hemp seed proteins were shown to have mixed-type inhibition, binding either to renin’s active site or to the enzyme-substrate complex [79]. Beyond direct enzyme inhibition, certain peptides can cross intestinal barriers intact and modulate renin at the transcriptional level. Pea protein hydrolysates reduced renal renin mRNA expression by approximately 50% in rats [125], demonstrating that some peptides can influence renin production rather than just enzyme activity.

#### 3.3.2. Structural Determinants of Renin Inhibition

The structural characteristics of peptides critically determine their renin-inhibitory potency. Net cationic charge in amino acid sequences facilitates renin inhibition through electrostatic interactions with the enzyme’s active site [83]. Molecular weight and amino acid positioning also influence activity. Peptides with low-molecular-weight hydrophobic residues at the N-terminus and high-molecular-weight residues at the C-terminus demonstrate enhanced renin inhibition. This structural arrangement may also provide resistance to endogenous proteases and facilitate intestinal absorption [126], enabling peptides to reach target tissues and inhibit renin mRNA expression [125]. Studies on flaxseed protein fractionation illustrate these principles. When broken down using various proteases and ultrafiltration, low molecular weight fractions (<1 kDa) showed moderate renin inhibitory effects with IC_50_ values ranging from 1220 to 2810 μg/mL [127].

#### 3.3.3. Renin-Inhibitory Activity of Plant Protein-Derived Peptides and Hydrolysate

Despite the therapeutic potential of renin inhibition, relatively few food-derived peptides exhibit strong renin-inhibitory activity, and most animal protein-derived peptides show no renin-inhibitory activity, with the exception of fish proteins [83]. Among plant-based sources, African yam hydrolysate (55.49%) [77], peanut hydrolysate (77.24%) [128], and Tartary buckwheat peptides such as LFFR and LGLLPYFR (IC_50_ values: 2909 and 9967 μg/mL, respectively) [129] are among the few with documented in vitro renin inhibition (Table 4). Some of these peptides have also demonstrated in vivo renin-inhibitory effects, including rapeseed-derived GHS (IC_50_ = 320 μg/mL) [76], mung bean peptide YADLVE (97% inhibition) [130], and hemp hydrolysate (>50% inhibition) [131].

As shown in Table 4, most renin-inhibitory activity studies use complex hydrolysates rather than defined peptide sequences, whereas ACE research typically focuses on short peptides. This may suggest that peptide mixtures can produce dual inhibition, with some peptides targeting ACE and others targeting renin. For instance, hemp hydrolysate showed 70% ACE inhibition and 35% renin inhibition, leading to a 38 mmHg reduction in blood pressure [79]. Similarly, pigeon pea hydrolysate showed 61.82% ACE inhibition and 14.28% renin inhibition, reducing blood pressure by 34.60 mmHg at a 100 mg/kg body weight (bw) dose [134]. Lima bean hydrolysate, with an ACE IC_50_ of 172.62 µg/mL and 31.73% renin inhibition, reduced systolic and diastolic blood pressure by 51% and 64%, respectively, at 15 mg/kg bw [105].

### 3.4. Alternative Antihypertensive Mechanisms for Plant-Derived Peptides

Aside from ACE and renin inhibition, bioactive peptides exert antihypertensive effects through multiple pathways. Arginine residues function as precursors of nitric oxide, promoting endothelium-dependent vasodilation. Kwak et al. (2013) reported that an 8-week supplementation with arginine-rich black soybean proteins reduced systolic blood pressure by 7.2 mmHg in pre-hypertensive subjects [136]. Peptides also provide cardiovascular protection through antioxidant pathways that mitigate oxidative stress [134]. The soybean tetrapeptide VHVV attenuated hypertension-induced renal damage in spontaneously hypertensive rats by activating the SIRT1-PGC1α/Nrf2 pathway, thereby restoring mitochondrial function and enhancing cellular defense systems [137]. These findings suggest that antihypertensive peptides may offer broader cardiovascular benefits beyond their direct enzyme-inhibitory effects.

Table 5 lists the antihypertensive studies with spontaneously hypertensive rats (SHRs). The moth bean hydrolysate at 5 mg/kg achieved a 30 mmHg SBP reduction [138], while 600 mg/kg of potato tuber hydrolysate produces a 60 mmHg mean arterial pressure (MAP) reduction [139]. These differences likely reflect variations in hydrolysate potency, peptide composition, and bioavailability, depending on the protein source and enzymatic treatment used. Furthermore, differences in the animal models used across studies may contribute to variability in effective dose ranges. To be able to obtain comparable data on the antihypertensive potential of peptides/hydrolysate derived from different protein sources, it is critical to measure the peptide and hydrolysate doses resulting in the same SBP or DBP drop or measure the blood pressure drop at the same dose using the same animal model, the same feeding method, and after the same feeding time.

Peptides that advanced to in vivo studies show variable in vitro potencies. Zein-derived LRP (IC_50_ = 0.104 µg/mL) achieved 15 mmHg blood pressure reduction [140], while wheat-derived IAP (IC_50_ = 0.814 µg/mL) produced a 60 mmHg reduction [141]. Rice-derived peptide TQYV, VNP, and VMP (IC_50_ values: 9.27, 2.10, 1.55 µg/mL, respectively) all progressed to in vivo validation with blood pressure reductions of 40, 29, 38 mmHg, respectively [142,143,144] (Table 5). This pattern indicates that factors beyond in vitro potency determine peptide advancement to physiological studies. These may include synthesis feasibility, stability, material availability, or cost. Publication bias toward positive results may also prevent a complete assessment of translation success rates. Clinical investigations represent an even smaller subset of the research pipeline. Limited clinical trials have confirmed the antihypertensive efficacy of plant proteins. A double-blind study by He et al. (2005) showed significant reductions in blood pressure among 139 participants who consumed 40 g of soybean protein over 12 weeks, particularly in subjects with moderate to severe hypertension [145]. The rice bran-derived peptide LRA showed potent antihypertensive activity in 44 participants during a 12-week supplementation study [146].

**Table 5:** In Vivo Antihypertensive Potentials of Plant-Derived Peptides: Protein Sources, Production Methods, and Blood Pressure Reduction.

Peptide Sequence	Protein Source	Production Method	Type of Animal	Route of Administration	Dose (mg/kg BW)	BP Drop (ΔmmHg)	Reference
YADLVE	Mung bean	Bromelain hydrolysis	SHRs	Oral Gavage	20	27 (SBP)	[130]
FDWLR	Walnut	Alcalase, pepsin, and pancreatin hydrolysis	Male SHRs	Oral Gavage	10	43.53 (SBP), 35.16 (DBP)	[38]
Crude hydrolysate	Moth bean	Alcalase hydrolysis	Dexamethasone-induced hypertensive rats.	Intraperitoneal	5	30 ± 2.37 (SBP)	[138]
LGF, GLFF	Moringa	Alcalase hydrolysis	SHRs	Oral Gavage	30	19.4, 18.2 (SBP) 12, 13.8 (DBP)	[90]
LDSPSEGRAPG	Wine lees	Flavourzyme hydrolysis	Male SHRs	Oral Gavage	10	19.0 ± 4.8 (SBP), 33.6 ± 3.6 (DBP)	[147]
MGR, HDCF	Garlic	Pepsin and pancreatin hydrolysis	SHRs	Oral	50	46.67, 49.33 (SBP), 32.33, 37.67 (DBP)	[148]
<1 kDa fraction (NL, QL, FL, HAL, AAVL, AKTVF, TPLTR)	Wheat bran	Alcalase hydrolysis	Male SHRs	Oral Gavage	100	35 (SBP)	[133]
Water-soluble peptide extract	Olive oil	Water extraction and FPLC fractionation	SHRs	Oral Gavage	0.425	20 (SBP)	[149]
Soy protein	Soybeans	Fermentation with *Lactobacillus rhamnosus*	Male SHRs	Oral Gavage	100	25 ± 4 mmHg (SBP), 40 ± 5 mmHg (DBP)	[150]
Crude hydrolysate	Lotus seeds	Protamex hydrolysis	SHRs	Oral Gavage	15	27.4 (SBP)	[91]
Crude hydrolysate	Kabuli chickpea	α-amylase, pepsin, and pancreatin hydrolysis	Male SHRs	Intragastrical	1200	61.41 (SBP)	[151]
Crude hydrolysate	Chickpea	Alcalase hydrolysis	Male SHRs	Intragastrical	50	47.35 (SBP)	[152]

Abbreviations: BW: body weight, SHRs: spontaneously hypertensive rats, SBP: systolic blood pressure, DBP: diastolic blood pressure, FPLC: fast protein liquid chromatography, ΔmmHg: change in blood pressure (negative values indicate BP reduction), MAP: mean artery pressure.

## 4. Bioavailability of Antihypertensive Peptides

To exert antihypertensive activity, the peptides must be bioavailable, that is, resistant to digestive peptidases and able to be transported through the brush border membrane intact and resistant to serum peptidases [153,154]. While in vitro tests measure direct enzyme inhibition under controlled conditions at optimal pH, these assays do not capture the complexity of digestion and systemic circulation. Consequently, many peptides with promising IC_50_ values are either degraded before absorption or fail to cross intestinal membranes intact, explaining why strong in vitro ACE inhibition rarely translates to measurable blood pressure reduction in vivo [155]. Although the bulk of bioavailability research has been conducted on animal-derived peptides, particularly from milk and fish, the same principles of digestive stability and intestinal transport are expected to apply regardless of protein source [40]. Developing strategies to increase peptide half-life is therefore essential for advancing plant-derived peptides as functional foods or therapeutic agents, with factors such as chain length, amino acid sequence, enzyme resistance, and hydrophilic/hydrophobic properties all influencing the likelihood of reaching the bloodstream in an active form [156].

### 4.1. Effects of Gastrointestinal Digestion on Antihypertensive Peptides and Protein Hydrolysates

Gastrointestinal (GI) digestion begins in the stomach, where acidic pH activates pepsin. Pepsin hydrolyzes peptide bonds between aromatic amino acids, breaking proteins into polypeptides and some free amino acids [157]. In the small intestine, pancreatic enzymes continue the breakdown of proteins/peptides. Trypsin cleaves internal bonds at lysine or arginine residues, producing smaller oligopeptides and activating other digestive enzymes. Chymotrypsin hydrolyzes bonds near aromatic or neutral amino acids, creating shorter oligopeptides [157]. Elastase breaks bonds of aliphatic amino acids (alanine, glycine, serine). Carboxypeptidases cleave from the C-terminal end. Carboxypeptidase A removes aromatic amino acids while carboxypeptidase B cleaves basic amino acids (arginine, lysine). Both produce free amino acids and smaller peptides [157].

The brush border of the intestinal lining represents the final stage of protein hydrolysis, where peptides break down into amino acids or smaller peptides for absorption [158]. The kinetics of human intestinal transport systems favor the absorption of di- and tripeptides over free amino acids [159]. Brush border peptidases and cytoplasmic peptidases have distinct substrate specificities [160]. Brush border peptidases, predominantly aminopeptidases, hydrolyze peptides at the N-terminal. These enzymes digest tripeptides most effectively, followed by tetrapeptides and oligopeptides, but have reduced efficiency with dipeptides and cannot hydrolyze peptides containing a C-terminal proline [157]. Cytoplasmic peptidases, located within enterocyte cytoplasm, primarily hydrolyze dipeptides and tripeptides [161]. Therefore, peptides with more than three amino acids can be further cleaved by GI digestion and/or brush border peptidases and cytoplasmic peptidases into shorter fragments that can be readily absorbed. This may partially explain why certain larger peptides and peptide mixtures in hydrolysates, which are typically >3 kDa, have greater in vivo antihypertensive potential [162]: they contain intermediate-length peptides that can undergo endogenous hydrolysis to generate resistant, absorbable peptides [163]. However, digestion does not always yield more active fragments. It was reported that rice protein hydrolysate generated by 2 h Alcalase hydrolysis had an IC_50_ of 0.14 mg/mL before digestion, which rose to 0.15 mg/mL after pepsin treatment, 0.20 mg/mL after pancreatin treatment, and 0.18 mg/mL after sequential pepsin-pancreatin treatment, indicating progressive loss of ACE inhibitory activity [143]. Similarly, Gu and Wu (2013) reported a significant loss of activity following in vitro gastrointestinal digestion of soybean protein hydrolysate [164].

### 4.2. Peptide Absorption Mechanisms

Peptides cross the intestinal epithelium through four routes: active transport, paracellular diffusion, transcellular passive diffusion, and transcytosis [165]. The primary mechanism involves active transport via the proton-coupled symporter PEPT-1, which represents the predominant route for di- and tripeptides (<500 Da). These transporters utilize proton electrochemical gradients, with sodium-proton exchangers maintaining pH balance [166]. Neutral peptides are absorbed more efficiently than charged peptides, with hydrophobic side chains increasing transporter interaction [167,168]. ACE inhibitor prodrugs, like Enalapril and Alacepril, utilize these transporters [169].

Alternatively, peptides can diffuse between enterocytes through tight junctions via paracellular transport, thereby avoiding proteolysis [170]. This route accommodates renin inhibitors [171] and casein-derived peptides [172]. For peptides with molecular weights ranging from 500 to 1600 Da, transport depends on hydrophilicity, size, flexibility, and the charge-to-mass ratio, with larger peptides requiring molecular flexibility to pass tight junctions [173]. Lipophilic peptides can also traverse epithelial membranes directly through transcellular passive transport, exemplified by Des-Asp-angiotensin I (DAAI) [174,175]. Finally, larger hydrophobic peptides undergo transcytosis, an energy-dependent vesicular transport mechanism from apical to basolateral membranes [176].

### 4.3. Peptide Fate in Circulation

Following absorption, peptides in the bloodstream may be susceptible to degradation by plasma peptidases, such as aminopeptidases and carboxypeptidases. They are found on the surfaces of mammalian tissues and within their cytoplasm, and are which are exopeptidases, which release amino acids from the ends of circulating peptides and proteins, thereby enhancing or reducing antihypertensive bioactivity [154,177]. The tripeptide Ile-Val-Tyr from wheat germ hydrolysate illustrates this process. Ile-Val-Tyr is hydrolyzed by plasma aminopeptidases to form the active dipeptide Val-Tyr. In spontaneously hypertensive rats, intravenous administration of Ile-Val-Tyr produced a longer blood pressure–lowering effect than VY, with Ile-Val-Tyr active for about 15 min and Val-Tyr active for about 5 min [178].

Once absorbed, peptides are rapidly cleared from circulation through two primary mechanisms: enzymatic breakdown by plasma peptidases or renal clearance, particularly for peptides with molecular weights below 30 kDa [179]. Circulating concentrations of most food-derived peptides are typically in the low nanomolar range in human blood [180]. For example, Val-Tyr reaches only 4 nM in human blood after oral doses of 30 mg [181], and Ile-Pro-Pro reaches 1 nM after consuming yogurt enriched with 20.4 mg of the tripeptide [182]. Peptides with specific structural features that confer exopeptidase resistance can reach higher blood concentrations [180]. Peptides containing post-translational modifications, such as hydroxyproline-containing dipeptides (Pro-Hyp) from collagen hydrolysates, can reach ~30 μM in blood [183], while pyroglutamyl peptides (pyroGlu-Pro) from grain hydrolysates reach 110 nM [184]. The position of proline within the peptide sequence also seems critical for bioavailability; peptides with proline at the N-terminus, such as Pro-Gly from elastin hydrolysate, reach concentrations of 18 μM [185], whereas peptides with proline at the C-terminus, such as Gly-Pro, do not significantly increase in blood despite being abundantly present in food sources [184]. Additionally, peptides with isomerized aspartyl residues, including those with D-aspartyl configurations and β-peptide bonds, demonstrate resistance to degradation [186].

Peptides that resist exopeptidase degradation by serum peptidases may escape rapid clearance and accumulate in various target organs, such as the kidneys, heart, lungs, and aorta, where they can exert their biological effects [187]. The systemic concentrations achieved by food-derived antihypertensive peptides are substantially lower than those of synthetic ACE inhibitors such as captopril, which has approximately 62% absolute oral bioavailability [188]. Despite these low circulating levels, food-derived peptides demonstrate antihypertensive effects in both human and animal studies. This paradox has led to the hypothesis that multiple mechanisms beyond systemic ACE or renin inhibition may contribute to their biological activity [180].

First, although some peptides demonstrate resistance to serum exopeptidases in vitro, they do not show a corresponding increase in blood concentrations when quantified in vivo, suggesting that they may be metabolized into other bioactive compounds with greater potency rather than remaining in their original form [189,190]. Second, many peptides originally identified as ACE inhibitors through in vitro screening may actually reduce hypertension through alternative mechanisms in vivo, such as direct relaxation of vascular smooth muscle or upregulation of ACE2, which degrades angiotensin II to generate the vasodilatory peptide angiotensin 1–7 [191]. Third, some peptides may exert effects locally in the intestine without requiring systemic absorption, acting on gut cells to modulate the microbiota [192], which has been linked to blood pressure regulation [193], or signaling through the vagus nerve to affect cardiovascular function in distant organs [194,195].

## 5. Challenges in Plant Protein-Derived Antihypertensive Peptides and Hydrolysates

Similar to the bioactive peptides and protein hydrolysates of animal origins, the production and applications of plant-derived antihypertensive peptides and hydrolysates face challenges like poor bioavailability, instability in the gut, and difficulty proving in vivo effects vs. in vitro results, bitter taste, low bioavailability, complex purification of specific bioactive peptides from mixtures, high production costs (especially enzymes/purification), inconsistency in quality, and strict regulatory standards for safety and efficacy.

### 5.1. Safety and Regulations

Plant-protein hydrolysates and peptides are generally considered safe for food and cosmetic use, especially when derived from proteins with a long history of safe consumption, but processing such as enzymatic hydrolysis and fermentation, generates peptides not typically seen in the gut, they are “new” to the body, potentially carrying allergic risks or unexpected toxicities, necessitating careful safety assessments [196,197]. In the United States, bioactive peptides/protein hydrolysates are not strictly regulated as “functional foods”; instead, they are classified as dietary supplements, conventional foods, or medical foods, based on the intended use and labeling claims. The US FDA allows the structure/function claims such as “supports healthy blood pressure,” relying on animal/in vitro data that require Significant Scientific Agreement (SSA) from experts, but requires strong human evidence/clinical trials for health claims, such as “prevents hypertension” and “reduces risk of CVD” [198]. In Canada, bioactive peptides, which do not fall into the drug’s category by definition, that are intended for functional food applications, do not need pre-market approval and regulatory amendment, but the claimed health benefits of bioactive peptides should not mislead the consumers, and the manufacturers should have substantial evidence (in-house) of health claim prior to submission of dossier to Health Canada [199]. In Europe, the bioactive peptides and hydrolysates are regulated under general food law, primarily falling under Novel Foods Regulation (EU) 2015/2283 if new, requiring European Food Safety Authority (EFSA) safety assessment, mainly based on the scientific substantiation and ability of an average consumer to understand the beneficial effects of the claims [200]. In addition, EFSA requires details relating to characterization of the peptides in terms of molecular weight distribution, number of peptides, amino acid composition, sequences, and length of the peptides, physical and chemical properties, manufacturing process, standardization of the product, conditions of use and stability of the peptides for the authorization of the health claims of bioactive peptides (EFSA NDA Panel, 2024) [201]. Without regulatory clarity and streamlined pathways, commercial development will remain hindered by compliance costs and market access uncertainty. Future research must establish standardized toxicological testing protocols tailored to bioactive peptides/hydrolysates; allergenicity prediction models based on peptide structure; and internationally recognized guidelines for minimum evidence requirements for health claims.

### 5.2. Bitterness of Bioactive Peptides and Hydrolysates

Bitterness poses a significant challenge to the commercial development of bioactive peptide products [202]. This bitter taste stems from hydrophobic amino acids released by endopeptidases during hydrolysis, with the amino acid sequence being the most important factor determining bitter taste threshold [203]. The intensity of bitterness is significantly affected by the protein source, the type of protease used, and the degree of hydrolysis [204,205]. Plant proteins, especially pea, soy, and rice, often yield more bitter peptides and hydrolysates than animal proteins due to inherent hydrophobic residues and secondary metabolites (saponins, polyphenols) [206,207]. Alcalase tends to create more bitterness than trypsin and Flavourzyme due to the exposure of hydrophobic amino acids [208]. Generally, the relationship between degree of hydrolysis and bitterness intensity is said to follow a bell-shaped curve, rising initially as bitter peptides accumulate, then declining as these peptides are further hydrolyzed into less bitter peptides [209,210]. Two strategies that can mitigate bitterness while preserving bioactivity are: (1) the strategic selection and application of exopeptidases (such as aminopeptidases or Flavourzyme), which can cleave bitter, hydrophobic peptides into smaller, less bitter fragments while generating bioactive sequences [209]; however, this approach may enhance or reduce the antihypertensive activity of the bitter peptides [211,212]. (2) Encapsulation, which can mask bitter taste by coating peptides with wall materials (such as maltodextrin, gum arabic, or β-cyclodextrin), thereby reducing exposure of hydrophobic groups [213]. While encapsulation adds processing steps, it may actually improve cost-effectiveness by simultaneously addressing multiple challenges: masking bitterness, protecting peptides during gastrointestinal transit to enhance bioavailability, and potentially enabling the use of less expensive crude hydrolysates rather than requiring the purification of specific non-bitter peptides [214,215]. Among encapsulation techniques, freeze-drying best preserves peptide stability and bioactivity due to its low-temperature processing [216], though its high operational cost, lengthy processing times, low throughput, and difficulty scaling to industrial volumes limit its commercial viability [217]. Spray drying, while potentially exposing peptides to thermal degradation, remains the most commercially successful method, and when paired with optimized wall materials, activity loss can be minimized to non-significant levels [218,219]. Spray freeze-drying has emerged as a promising technology by combining the atomization of liquid sample into small droplets, flash freezing and low-temperature freeze-drying to offer improved powder properties and better preservation of bioactivity [220], though it has high capital and operational costs, and has not yet achieved widespread commercial adoption [221].

### 5.3. Enhancement of Peptide Stability and Bioavailability

As demonstrated throughout this review, many peptides with high in vitro enzyme-inhibitory activity fail to achieve therapeutic effects in blood pressure reduction in vivo due to degradation by gastrointestinal proteases and poor intestinal absorption. The therapeutic efficacy of plant-derived ACE-inhibiting and renin-inhibiting peptides depends on their delivery to target sites, maintenance of structural integrity throughout gastrointestinal transit, and attainment of bloodstream concentrations sufficient to inhibit the enzymes.

Encapsulation with food-grade polymers remains the primary approach for protecting bioactive peptides during processing, storage, and gastrointestinal transit. For instance, it was reported that stone fish (*Actinopyga lecanora*) derived bioactive peptides encapsulated into chitosan nanoparticles showed significantly higher blood pressure-lowering effect than the free peptides on a spontaneously hypertensive rat model [222]. The encapsulation techniques reported for bioactive peptide/hydrolysate protection include spray drying, ionic gelation, liposomes, emulsification, and coacervation [223]. Spray drying is cost-effective but exposes peptides to high temperatures, which may compromise their bioactivity [166]. The coacervation involves phase separation of oppositely charged biopolymers (e.g., gelatin-gum arabic, whey protein-pectin) to form peptide-loaded microcapsules, but often results in droplet instability during formation and is poorly scalable to industrial volumes [224]. Liposomes prepared from food-grade lecithin (soy, sunflower, or egg phospholipids) can encapsulate peptides and provide protection from enzymatic degradation [225]. However, liposomes face significant practical challenges: they are physically unstable during storage (leading to vesicle fusion and leakage), have low encapsulation efficiency for small hydrophilic peptides, and require cholesterol to improve stability, which may be undesirable for most consumers due to dietary restrictions or negative health perceptions [225]. Hydrogel-based systems using food-grade gelling agents can provide some pH-responsive protection [226,227]. Alginate beads formed by ionotropic gelation with calcium ions can protect peptides during gastric transit and release them in the small intestine [228,229]. Further research is needed to better understand their performance under physiological conditions before reliable application in food systems can be achieved. Several commercial products already incorporate protein hydrolysates and bioactive peptides in encapsulated forms to improve stability and bioavailability, such as Designs for Sport’s Pro-formance Peptides and Integrative Peptides’ BPC-157 Pure.

Co-consumption of certain dietary components can, in theory, influence peptide stability and absorption [230,231]. For example, it was found that protein and carbohydrate (including dietary fiber) in the food matrix enhanced the stability and bioavailability of peptides [230], while polyphenols interact with bioactive peptides in the food matrix, hindering the digestion and absorption of the peptides [232]. Although the concept of food matrix design to enhance bioavailability has precedent in other bioactive compounds, no systematic studies have demonstrated that strategic food matrix design or co-consumption patterns meaningfully improve peptide bioavailability or antihypertensive efficacy in humans. This represents a significant research gap.

## 6. Conclusions and Future Perspectives

Plant protein-derived antihypertensive peptides and hydrolysates consistently demonstrate strong ACE- and renin-inhibitory activity in vitro across diverse sources, from legumes to oilseeds, cereal grains, fruits, and vegetables. However, their in vivo efficacy is often limited by the stability and bioavailability of the effective peptides against endogenous digestion, which can compromise absorption and ultimately reduce antihypertensive activity. Bridging this gap will require a shift from isolated in vitro studies to integrated approaches that address the full complexity of peptide delivery in real-world food applications. Various encapsulation techniques have demonstrated potential as peptide protection strategies, but each has its drawbacks. Most critically, there is a severe lack of systematic bioavailability studies comparing different food-grade delivery systems in human subjects using validated pharmacokinetic endpoints (intact peptide appearance in plasma, ACE activity in serum, blood pressure reduction). The vast majority of encapsulation studies measure in vitro release profiles using simulated gastric and intestinal fluids, but do not verify whether peptides reach target tissues in bioactive form or whether encapsulation provides any clinical benefit over unprotected peptides. Furthermore, most encapsulation methods described in the literature remain at laboratory scale (milligram to gram quantities); scale-up to food manufacturing volumes (kilograms to metric tons) has not been demonstrated for most systems, and techno-economic analyses showing manufacturing costs at commercial scale are rarely provided.

Three parallel research priorities stand out. First, well-designed human clinical trials are needed to rigorously demonstrate the antihypertensive efficacy of peptides or peptide-rich hydrolysates using validated endpoints such as ambulatory blood pressure monitoring and vascular function assessments in hypertensive populations. Second, a systematic investigation of peptide–food matrix interactions is essential, as protein hydrolysates are rarely consumed in isolation but rather as ingredients in complex formulations, including beverages, baked goods, other processed foods, and supplements, where interactions with lipids, carbohydrates, minerals, and processing-derived compounds may alter peptide stability, release kinetics, and bioavailability. Third, scalable protection strategies that reduce peptide degradation by endogenous proteases while remaining economically viable at commercial manufacturing scales must be developed. Even if these scientific challenges are addressed, the broader adoption of plant-derived antihypertensive peptides will depend on clear regulatory frameworks defining standards for safety, efficacy, and health claims, supported by robust clinical evidence. Ultimately, advancing these peptides as functional ingredients will require integrated solutions that balance bioavailability, efficacy, and industrial feasibility.

## Figures and Tables

**Table 1:** Classification of hypertension according to blood pressure levels [14].

Category	SBP (mmHg)	DBP (mmHg)
Normal	<120	and < 80
Elevated/Prehypertension	120–129	and <80
Stage 1 Hypertension	130–139	and/or 80–89
Stage 2 Hypertension	140–159	and/or 90–99
Stage 3 Hypertensive crisis	≥160/180	and/or ≥100

**Table 2:** Antihypertensive Drugs: Classes, Mechanisms and Side Effects [34,35].

Drug Class	Drug Names	Mechanism of Action	Side Effects
ACE Inhibitors	Lisinopril, Enalapril, Ramipril, Perindopril, Captopril	Inhibit ACE → ↓ angiotensin II + ↑ bradykinin levels → vasodilation	Dry cough, Angioedema, hyperkalemia, acute kidney injury, Anemia, hypotension
Angiotensin Receptor Blockers (ARBs)	Losartan, Valsartan, Irbesartan, Candesartan, Olmesartan	Block AT_1_ receptors → prevent angiotensin II effects → vasodilation	Hyperkalemia, acute kidney injury, cough
Calcium Channel Blockers	DHPs: Amlodipine, Nifedipine Non-DHPs: Verapamil, Diltiazem	Block L-type Ca^2+^ channels → smooth muscle relaxation + ↓ cardiac contractility	DHPs: Peripheral edema, flushing, headache, tachycardia Non-DHPs: Bradycardia, constipation (verapamil) All: Gingival hypertrophy
Diuretics-Loop	Furosemide, Bumetanide, Torsemide	Inhibit Na^+^/K^+^/2Cl^−^ cotransporter (NKCC) in the loop of Henle	Hyponatremia, hypokalemia, hypocalcemia, hypomagnesemia, hyperuricemia, hyperglycemia, hyperlipidemia, ototoxicity
Diuretics-Thiazides	Hydrochlorothiazide, Chlorthalidone, Indapamide	Inhibit Na^+^/Cl^−^ cotransporter in the distal tubule	Hyponatremia, hypokalemia, hypercalcemia, hypomagnesemia, hyperuricemia, hyperglycemia, hyperlipidemia
Diuretics-K^+^ Sparing	Spironolactone, Eplerenone, Amiloride	Spironolactone/Eplerenone: Aldosterone receptor antagonists Amiloride: ENaC channel blocker	Spironolactone: Gynecomastia, sexual dysfunction, menstrual irregularities All: Hyperkalemia
β-Blockers	Non-selective: Timolol, Propranolol β_1_-selective: Metoprolol, Atenolol Vasodilating: Carvedilol, Nebivolol	Block β-adrenergic receptors → ↓ heart rate, ↓ cardiac output, ↓ renin release	Bronchospasm, masking hypoglycemia, glucose intolerance, vivid dreams, depression, sexual dysfunction, cold extremities, fatigue
Renin Inhibitors	Aliskiren	Direct renin inhibition → ↓ angiotensinogen to angiotensin I conversion	Headache, diarrhea, dizziness, fatigue

Abbreviations: ACE, angiotensin converting enzyme; ARBs, angiotensin receptor blockers; AT_1_, angiotensin II type 1 receptor; DHPs, dihydropyridines; AV, atrioventricular; NKCC, Na^+^/K^+^/2Cl^−^ cotransporter; ENaC, epithelial sodium channel. Mechanistic pathways (→), Increases (↑), and decreases (↓).

**Table 3:** In Vitro ACE Inhibitory Activities of Plant-Derived Peptides: Sources, Production Methods, and IC_50_ Values.

Peptide Sequence	Protein Source	Production Method	IC_50_ for ACE Inhibition *	Reference
FDWLR	Walnut	Alcalase, pepsin, and pancreatin hydrolysis	8.02 µg/mL	[38]
PW, VTLL, LPGP, SPGTAF	Maize germ	Flavourzyme hydrolysis	446 ± 21 µg/mL, 996 ± 4 µg/mL, 788 ± 4 µg/mL, 1339 ± 35 µg/mL	[58]
YGIKVGYAIP	Palm kernel cake	Papain hydrolysis	1.08 μg/mL	[59]
SAPPP	Quinoa bran	Papain hydrolysis	510 µg/mL	[86]
KSVLLKF	Broccoli	Pepsin and pancreatin hydrolysis	0.129 μg/mL	[87]
VVVPQN	Camellia seeds	Neutral protease, alkaline protease, papain, and trypsin hydrolysis	130 μg/mL	[88]
IWHHTFYNELR LGF GLFF	Moringa	Alcalase hydrolysis	1057 µg/mL 97.3 ± 43.6 µg/mL 149.6 ± 19.3 µg/mL	[89,90]
WSF, FGFL	Lotus seeds	Protamex hydrolysis	34.86 µg/mL, 36 µg/mL	[91]
TLVY LLVY	Beefsteak plant seeds	Thermolysin hydrolysis	15.33 μg/mL, 22.29 μg/mL,	[92]
EVPQAYIP	Oil palm kernels	Papain hydrolysis	94.12 μg/mL	[93]
KDFPPR, VVPPGHPF, DTFPYPR	Black soybean	Alcalase hydrolysis	7910 μg/mL, 12130 μg/mL, 10690 μg/mL	[94]
SNHANQLDFHP, PVQVLASAYR	Pumpkin seeds	Neutrase 5.0 BG hydrolysis	220.1 μg/mL, 100.1 μg/mL	[95]
VNDYLNW	Chinese nutmeg yew	Alkaline protease hydrolysis	190.1 μg/mL	[96]
HWS, VLSGF	Peony seeds	Neutral protease hydrolysis	640 ± 16 μg/mL, 328 ± 40 μg/mL	[97]
ERFNVE, TELVLK, MELVLK, FDDKLD	Mulberry leaf	Flavourzyme hydrolysis	2650 µg/mL, 980 µg/mL 1900 µg/mL, 700 µg/mL	[98]
DLLGCS	Corn gluten meal	Alcalase hydrolysis	23.0 µg/mL	[99]
PLLK, PPMWPFV	Millet bran	Papain, Alcalase, and trypsin hydrolysis	298 µg/mL, 364 µg/mL	[100]
LGAVPPRY, IARDSAAVF, VYLAELHF	Taiwan red quinoa	Thermolysin hydrolysis	25.6 µg/mL, 53.5 µg/mL, 115 µg/mL	[101]
QYVPF, GYHGH	Oat bran	Cellulase, papain, and flavourzyme hydrolysis	136.9 µg/mL, 154.4 µg/mL	[102]
IIPNEVY, ITPPVMLPP	Green coffee	Alcalase and thermolysin hydrolysis	48.73 μg/mL, 38.92 μg/mL	[103]
DLSSAP	Basil leaves	Pepsin and trypsin hydrolysis	2.80 μg/mL	[104]
Crude hydrolysates	Peanut	Alcalase hydrolysis	5450–7400 µg/mL	[57]
Hydrolysate (Fraction <5 kDa)	Peanut	Alcalase hydrolysis	850 µg/mL	[57]
Hydrolysate (Fraction <3 kDa)	Lima bean	Pepsin and pancreatin hydrolysis	2.69 μg/mL	[105]
Crude hydrolysate	Lupin	Alcalase hydrolysis	3210 ± 60 µg/mL	[106]
Crude hydrolysate	Job’s tears	Pronase hydrolysis	52 µg/mL	[107]
Crude hydrolysate	Tarwi	PC7 and Alcalase hydrolysis	13.5 ± 1.1 μg/mL	[108]
Hydrolysate (Fraction <1 kDa)	Peach Kernel	Alcalase hydrolysis	780 μg/mL	[109]
Hydrolysate (Fraction <3 kDa)	Black sesame	Flavourzyme hydrolysis	150 ± 30 µg/mL	[110]

* Abbreviations: IC_50_: Concentration required for 50% enzyme inhibition (lower values indicate stronger inhibition). All IC_50_ values in Table 3 have now been standardized to μg/mL for improved comparability.

**Table 4:** In Vitro Renin Inhibitory Activities of Plant-Derived Peptides: Sources, Production Methods, and IC_50_ Values.

Peptide/Hydrolysate	Protein Source	Production Method(s)	Renin IC_50_ or Inhibition (%)	Reference
GHS	Rapeseed	Enzymatic hydrolysis (pepsin + pancreatin)	IC_50_: 320 ± 10 µg/mL	[76]
LFFR, LGLLPYFR	Tartary buckwheat	Enzymatic hydrolysis (pepsin + trypsin)	IC_50_: 2909 μg/mL, IC_50_: 9967 μg/mL	[129]
YADLVE	Mung bean	Enzymatic hydrolysis (bromelain)	97 ± 3.06%	[130]
FNLPILR	Amaranth	Enzymatic hydrolysis (Alcalase)	IC_50_: 350 µg/mL	[132]
LGF, GLFF	Moringa	Enzymatic hydrolysis (Alcalase)	IC_50_: 630 ± 295 µg/mL, IC_50_: 1351 ± 39 µg/mL	[90]
Crude hydrolysates	Peanut	Alcalase hydrolysis	32.6–54.9%	[57]
Hydrolysate (Fraction <5 kDa)	Peanut	Alcalase hydrolysis	1780 µg/mL	[57]
Crude Hydrolysate	Hemp	Enzymatic hydrolysis (pepsin + pancreatin)	35%	[79]
Crude hydrolysate	African yam bean	Enzymatic hydrolysis (Alcalase)	55.49%	[77]
Crude hydrolysate	Peanut	Enzymatic hydrolysis (neutrase + protamex) + high-pressure microfluidization	77.24 ± 3.81%	[128]
Crude hydrolysate	Hemp	Enzymatic hydrolysis (Alcalase)	>50%	[131]
Crude hydrolysate	Hemp	Enzymatic hydrolysis (pepsin)	IC_50_: 79 µg/mL	[131]
Hydrolysate <1 kDa fraction (NL, QL, FL, HAL, AAVL, AKTVF, TPLTR)	Wheat bran	Enzymatic hydrolysis (Alcalase)	75.19 ± 1.75%	[133]
Crude hydrolysate	Pigeon Pea	Enzymatic hydrolysis (thermoase)	IC_50_: 570 µg/mL	[134]
Crude hydrolysate	Pigeon pea	Enzymatic hydrolysis (pepsin + pancreatin)	14.28%	[134]
Hydrolysate (Fraction <3 kDa)	Lima bean	Enzymatic hydrolysis (pepsin + pancreatin)	31.73%	[135]

Abbreviations: IC_50_: Concentration required for 50% enzyme inhibition (lower values indicate stronger inhibition). Where available, IC_50_ values (µg/mL) are reported. When only percentage inhibition was provided in the original study, the value is presented as a percentage. All IC_50_ values have been standardized to µg/mL for comparability.

## Data Availability

No new data were created or analyzed in this study. Data sharing is not applicable to this article.
